# Urinary Excretion of Cyanuric Acid in Association with Urolithiasis: A Matched Case-Control Study in Shanghai Adults

**DOI:** 10.3390/ijerph19148726

**Published:** 2022-07-18

**Authors:** Feifei Huang, Qilai Long, Shaojie Liu, Yanyun Chen, Yifei Wang, Hangwei Wang, Ruihua Dong, Jianming Guo, Bo Chen

**Affiliations:** 1 Key Lab of Public Health Safety of the Ministry of Education, School of Public Health, Fudan University, Shanghai 200032, China; 19211020098@fudan.edu.cn (F.H.); liushaojie@fudan.edu.cn (S.L.); 20211020050@fudan.edu.cn (Y.W.); 20211020146@fudan.edu.cn (H.W.); ruihua_dong@fudan.edu.cn (R.D.); 2 Department of Urology, Zhongshan Hospital, Fudan University, Shanghai 200032, China; long.qilai@zs-hospital.sh.cn (Q.L.); chen.yanyun@zs-hospital.sh.cn (Y.C.)

**Keywords:** cyanuric acid, urolithiasis, urinary excretion, chlorinated isocyanuric acid disinfectants

## Abstract

Melamine (MEL) has raised human concern since the 2008 milk scandal. Co-exposure to MEL and one of its analogues, cyanuric acid (CYA), has been reported to have a synergistic effect on promoting urolithiasis. However, few epidemiological studies have reported urolithiasis in association with exposure to CYA based on our knowledge. We therefore conducted a case-control study to investigate whether cases of urolithiasis had higher excretion of urinary CYA than the controls. Spot urine samples from 70 adult cases and first-morning urine samples from 70 controls (matched by age and sex) were collected for the measurement of MEL, CYA, and other two analogues in urine. The case group also had 2.81-fold higher concentration of urinary CYA than the control group (34.87 versus 12.43 ng/mL, *p*-value < 0.001). Multivariate conditional logistic regression models adjusting potential confounders of personal characteristics identified the risk factor of urinary CYA as a continuous variable with odds ratio (OR) (95% confidence interval, 95%CI) of 1.11 (1.02–1.21) (*p*-value = 0.021) and having meals at restaurants with OR of 5.71 (1.01–32.31) (*p*-value = 0.049). Compared to the participants having the lowest quartile of CYA concentration in urine, participants at the second, third, and fourth quartile groups had ORs of 13.94, 83.69, and 118.65 with *p*-values of 0.004, <0.001, and <0.001, respectively. The high excretion of urinary CYA in urolithiasis cases might be the sign of stones in patients consisting of CYA, then proving the attribution of CYA exposure in the etiology of urolithiasis. These findings are important since CYA is a degraded by-product of chlorinated isocyanuric acid disinfectants, which are widely used in daily life not only in swimming pool water but also in other scenarios, such as serving as anti-pandemic disinfectants. Risk assessment of CYA serving as a by-product of disinfectants needs to be conducted in future studies.

## 1. Introduction

Melamine (MEL) has raised human concern since the 2007 pet food scandal in North America [1,2] and the 2008 milk scandal in China [3,4]. During the scandals, cases were found to suffer from urolithiasis, which was proven by later experiments in animals and epidemiological surveys in humans [5,6,7,8]. The crystals or stones in the urinary tract by exposure to MEL was reported to mainly consist of complexes of MEL with uric acid or cyanuric acid (CYA) [9,10]. Animal studies reported a strong synergistic effect on urolithiasis in the event of co-exposure to both MEL and CYA [11,12,13].

It is generally thought that urolithiasis caused by MEL was associated with scenarios of high levels of exposure, such as adulteration of milk powders [14]. However, several case-control studies in Taiwan reported higher excretion of urinary MEL in adult urolithiasis cases and therefore suspected the attribution of low levels of exposure to MEL [15,16]. Several cross-sectional studies in mainland China, Taiwan, and America also reported renal dysfunction in association with low levels of exposure to MEL [5,17,18,19]. Considering the stronger synergistic effect (12–20-fold higher, as suggested by Xie et al. [20] and Gamboa da Costa et al. [21]) of co-exposure to MEL and CYA compared to single exposure to MEL, we might also suspect urolithiasis in association with low levels of exposure to CYA. However, little evidence has been reported in the literature of human data.

More importantly, such suspicion could be more reasonable since the literature has reported a higher level of exposure to CYA than MEL in both the general population and in children [22]. The pollution examination of environmental media, including food, water, soil, and sludge, etc., also reported a typical 2–3-fold higher level of exposure to CYA than MEL [23,24].

Because of its ability to stabilize chlorine [25], CYA is widely used as a raw material for organic chlorine-containing disinfectants in industry, which are commonly applied to the disinfection of drinking water, swimming pool water, medical and health institutions, aquaculture, public health places, and industrial water treatment and so on [26,27,28]. Two of the most common chlorinated isocyanuric acid disinfectants are sodium dichloroisocyanurate and trichloroisocyanuric acid, both of which can be degraded in water to produce CYA and may become an important source of pollution to the environment [29,30]. During the coronavirus (COVID-19) pandemic, chlorine disinfectants were used as a routine strategy to counteract the possible contamination from the virus in the environment, especially in China [31]. Such massive application of disinfectants may increase the background pollution of CYA in the environmental media and therefore raise greater concern of urolithiasis [32].

In this study, we conducted a case-control survey to investigate whether cases of urolithiasis had higher excretion of urinary CYA than the matched controls. Since CYA in the human body has been reported to be excreted mainly through urine in prototype [33], a higher excretion of urinary CYA in cases than the controls may predict a higher exposure to CYA in cases and therefore support the hypothesis of urolithiasis in association with CYA exposure.

## 2. Materials and Methods

### 2.1. Study Subjects

This study recruited 70 adult patients who were diagnosed with urolithiasis by B-ultrasound in the urology department at Zhongshan Hospital, affiliated to Fudan University, from September 2020 to January 2021. We excluded the patients with medical history of chronic urinary tract infection, renal failure, chronic diarrhea, gout, renal tubular acidosis, hyperthyroidism, tumor, and other diseases. Anyone that had taken drugs or health products in the past 6 months, such as diuretics, potassium preparations, vitamin D tablets, and calcium tablets, were also excluded.

Applying the same exclusion criterion and a simple random sampling method, we selected controls (without history of urolithiasis and no clinical finding of stones confirmed by B-ultrasound) from the Shanghai Suburban Adult Cohort and Biobank. The cohort profile has been described in the previous study [34]. Briefly, the cohort conducted a baseline survey on 44,887 participants in seven communities from 6 April 2016 through 31 October 2017. Seventy participants from one community were randomly selected to serve as controls matched (1:1) with cases by age (±3 years) and sex.

### 2.2. Questionnaire Survey and Sample Collection

All participants were interviewed by trained investigators using structured questionnaires, including personal information (age, gender, body weight and height, labor intensity, and medical history of having stones in the urinary system) and behaviors (cigarette-smoking and alcohol-drinking behavior, fluid intake, swimming, and eating behavior). The participants’ frequency of having meals at the canteen was divided as “barely” (≤1~2 times/week) and “frequently” (≥3~4 times/week), and the frequency of having meals at restaurants as “barely” (≤1~3 times/month) and “frequently” (≥1~2 times/week). Participants who had swimming behavior in the swimming pool ≥1 time/summer were defined as “yes”. Body weight and height were used to calculate the body mass index (BMI).

Spot urine samples from cases and first-morning urine samples from controls were collected for the measurement of urinary pH, MEL, and its analogues. Indexes of kidney function (serum urea nitrogen, serum creatinine, and serum uric acid) were from the hospital for cases and from baseline survey record for controls.

### 2.3. Measurement of Melamine and Its Analogues in Urine

MEL and its three analogues (ammeline (AMN), ammelide (AMD), and CYA) were simultaneously detected by ultra-performance liquid chromatography tandem mass spectrometry (UPLC-MS/MS). The detailed detection methodology of MEL and its analogues has been described in the previous study [35]. Briefly, 100 μL internal standard solution and 3.9 mL acetonitrile were added into 1 mL urine sample. After vortex, sonication, and centrifugation, the urine sample was firstly loaded into the activated mixed cation exchange (MCX) solid-phase extraction column for the enrichment of MEL, AMN, and AMD and then into mixed anion exchange (MAX) solid-phase extraction column for the enrichment of CYA. The extracted solution was eluted with 5% ammoniated methanol or 2% formic acid methanol, dried under nitrogen at 40 °C, and then separated by amide chromatographic column (2.1 mm × 100 mm, 1.7 μm). Tandem mass spectrometer was used to perform qualitative and quantitative analysis in the simultaneous scanning mode of positive and negative ions. MEL, AMN, AMD, and CYA had limits of detection (LOD) of 0.03, 0.04, 0.04, and 0.05 ng/mL and limits of quantification (LOQ) of 0.11, 0.12, 0.14, and 0.15 ng/mL, respectively.

### 2.4. Statistical Analyses

The population of all participants in this study had the detection prevalence of 76.42%, 41.43%, 97.86%, and 98.57% for MEL, AMN, AMD, and CYA, respectively. One-half of the LOD values were assigned to the participants with MEL or its analogues less than LOD. Univariate and multivariate conditional (matched) logistic regression models were established to explore the association of urolithiasis with personal characteristics, kidney function indexes, and the exposure to MEL and its analogues. The adjusted confounders in the multivariate models were those differently distributed in cases and controls with *p*-value of <0.05 and those being reported to be positively associated factors (fluid intake and BMI). Since the continuous value of CYA presented a remarkable significance in both univariate and multivariate models, we further explored its association with urolithiasis by dividing its value into two (using median cut-point) or four groups (using quartile cut-point). Such strategy of turning the continuous variable into a categorical variable presented a much more remarkable significance for the association between urolithiasis and the exposure to urinary CYA. All analyses were performed using SAS software version 9.4 (SAS Institute Inc., Cary, NC, USA). A *p*-value of <0.05 was considered statistically significant.

## 3. Results

Table 1 presents the personal characteristics of adult patients with urolithiasis and the controls. Compared to the control group, the case group had higher prevalence of labor intensity, alcohol drinking, and swimming behavior; ate more frequently at either canteens or restaurants; and had higher concentrations of serum creatinine and uric acid. The case group also had 2.81-fold higher concentration of urinary CYA than the control group (34.87 versus 12.43 ng/mL, *p*-value < 0.001).

Table 2 presents the results of conditional logistic regression analyses by comparing cases with controls. Univariate models identified risk factors of drinking alcohol, having meals at canteens or at restaurants, and having higher concentrations of serum creatinine, serum uric acid, and urinary CYA (*p*-value < 0.05). Labor intensity and swimming frequency in summer were also risk factors with odds ratios (ORs) close to the statistical significance (*p*-value = 0.056 and 0.069, respectively). Without considering MEL and its analogues, multivariate models after adjusting significant variables of personal characteristics maintained the significance of ORs with frequently having meals at restaurants, serum creatinine, and serum uric acid. When including the variables of MEL or its analogues, multivariate models adjusting significant variables of personal characteristics only identified the risk factor of CYA with OR (95% confidence interval, 95%CI) of 1.11 (1.02–1.21) (*p*-value = 0.017).

Table 3 presents the results of stratified CYA concentration in association with the risk of urolithiasis by conditional logistic regression models. Either stratified by median or quartile values, higher concentration of urinary CYA was associated with higher ORs. Compared to the participants of having lowest quartile of CYA concentration in urine, participants at the second, third, and fourth quartile groups had ORs of 13.94, 83.69, and 118.65 with *p*-values of 0.004, < 0.001, and < 0.001, respectively.

## 4. Discussion

In this study, we found a significantly higher excretion of urinary CYA in cases of urolithiasis than in the controls. The logistic regression model presented an OR of 1.11 for CYA as a continuous variable after adjusting confounders, which meant a 1 ng/mL increase of urinary CYA would lead to a 1.11-fold higher risk of having urolithiasis. Such an effect was even more remarkable when we turned the urinary CYA into a categorical variable using either median or quartile cutoff points. The exposure level of urinary CYA was similar to the findings in U.S. children [5]. This is the first study, based on our knowledge, reporting epidemiological data of urolithiasis in association with exposure to CYA.

The results need to be carefully explained. General knowledge on the toxicokinetics of CYA in the human body is that it is rapidly absorbed after administration and eliminated unchanged via the urine with an elimination half-life of about 3 h [36]. Based on such knowledge, one cannot attribute the results of higher excretion of urinary CYA to the CYA exposure before the appearance of stone nidus in urolithiasis cases. The case-control design of this study might occasionally find a higher excretion of urinary CYA in cases than in controls due to the temporary exposure to CYA in the sampling day.

However, in a case study of the 2008 milk scandal, Ching-Wan Lam et al. [37] found a higher excretion of urinary MEL in infants with urolithiasis even after at least 10 days of stopping the consumption of MEL-tainted milk products. The researchers also found the excretion of MEL was positively correlated to the size of kidney stone. Since MEL and CYA share similar toxicokinetics in the human body, one may deduce that the excretion of CYA could be also prolonged in the case of the urinary tract having stones consisting of CYA [33]. It should be noted that the case study by Lam et al. did not find a higher excretion of urinary CYA, which was different from the results of urinary MEL. How can such a contradiction between the MEL and CYA in their paper be explained? Basically, animal data in the literature presented that MEL-induced stones or crystals were MEL-urate complexes (formed by co-exposure to MEL and uric acid) [38] or MEL-cyanurate complexes (formed by co-exposure to MEL and CYA) [39,40]. Literature suggested that the urolithiasis cases in infants during the milk scandal consisted of mainly MEL-urate complex rather than MEL-cyanurate complex since infants took milk as the staple food, and few contaminations of CYA were found in milk products [41]. However, the adult urolithiasis cases in this study might consist of both MEL-cyanurate complex and MEL-urate complex since the general population was usually exposed to a 2–3-fold higher level of CYA than MEL. The key question to be answered is whether the MEL-cyanurate complex-related urolithiasis will prolong the toxicokinetics of MEL and CYA in human body. If the answer is “yes”, the findings that a higher excretion of urinary CYA in urolithiasis cases might be a direct sign of stones consisted of CYA, then proving the attribution of CYA exposure in the etiology of urolithiasis.

Besides the finding of a higher excretion of urinary CYA in cases, another finding might also suggest the importance of CYA exposure in the etiology of urolithiasis: swimming frequency presents an OR of 7.00, which is close to being significant (*p*-value = 0.069). The lack of significance might be associated with the small sample size since only 10 participants reported a frequency of ≥1 time/summer. Water in swimming pools is usually disinfected using chlorinated isocyanuric acid disinfectants, which can be degraded to produce CYA [42]. Zhu et al. [43] reported a high level 1.5 × 10^7^ ng/L in swimming pool water, which was around 1.5 × 10^5^-fold higher than that in bottled water (median value: 98 ng/L). Swimming behavior may be a remarkable source of CYA exposure and needs to be assessed for health concern. However, few studies in the literature conducted such a risk assessment.

One other interesting finding was that urinary MEL in cases was not associated with urolithiasis. The case group even had a lower excretion of urinary MEL than the control group although the *p*-value was not significant. If the deduction of CYA in association with urolithiasis and the stones in the cases were consisted of MEL-cyanurate complex, then how can the contradiction between the findings of MEL and CYA be explained? In this study, the cases provided spot urine samples, while the controls provided first-morning urine samples. There was a 1.3-fold higher excretion of urinary MEL in first-morning urine (12.07 ng/mL) than that in spot urine (mean value: 9.25 ng/mL) in our unpublished data of a panel study, which compared MEL concentration in all urine samples of graduate students collected in 24 h. A similarly designed case-control study by Liu et al. [15] found a higher excretion of urinary MEL in cases than the controls. However, Liu et al.’s study did not present the data of CYA. The etiology of urolithiasis by both MEL and CYA based on Liu et al.’s study and this study need to be confirmed in future studies.

Another finding in this study might also suggest the etiology of MEL: having meals at the canteens or restaurants showed as being risk factors of urolithiasis in univariate logistic regression models, and having meals at restaurants remained significant even after adjusting the confounders. The reason might be attributed to the potential exposure to MEL at canteens and restaurants since these places likely use tableware made by MEL-formaldehyde resin, which was reported to release MEL to food [44,45]. Other reasons for eating at restaurants being found as a risk factor might be attributed to the food pattern [17].

The urolithiasis cases in this study present a higher concentration of both serum creatine and serum uric acid (Table 1). Similar results on these two indexes of kidney function were also reported in the case studies of the 2008 milk scandal and in the animal studies [9,37]. This study also presented a possible impact of higher labor intensity and alcohol-drinking behavior on increasing the risk of having urolithiasis (Table 1 and Table 2). People who are more labor-intensive sweat heavily, which leads to loss of body fluids, thereby concentrating the urine. The concentration of lithogenic substances in the urine is therefore increased and leads to the formation of stones. For alcohol-drinking behavior, most studies in the literature reported an inverse association between alcohol consumption and kidney stones [46,47,48], which were in contrast to the findings of the current study. Such inconsistence between this study and the literature may be due to the small sample size or the misclassification of alcohol behavior.

This study has several major limitations need to be addressed. Firstly, the sample size was small. Secondly, the control group was from the community but not from the same hospital of the case group, which might lead to selection bias. Thirdly, urine samples from controls were first-morning urine samples, while that from cases were not, which also lowered the comparability between cases and controls. However, an experiential higher level of excretion in first-morning urine than that in spot urine strengthened the significance of urinary CYA since the spot urine samples from the cases in this study already presented a 2.81-fold higher concentration than that in first-morning urine samples in the controls.

## 5. Conclusions

We found a significantly higher excretion of urinary CYA in urolithiasis cases than that in controls, which might suggest the importance of exposure to CYA in the etiology of urolithiasis. Such a finding is important since CYA is a degraded by-product of chlorinated isocyanuric acid disinfectants, which are widely used in daily life not only in swimming pool waters but also in other scenarios, such as serving as anti-pandemic disinfectants. Risk assessment of CYA serving as a by-product of disinfectants needs to be conducted in future studies. The finding of CYA exposure in association with urolithiasis may also have clinical implications since patients having stones composed of CYA may have different features in clinical symptoms and biochemistry, which may benefit the diagnosis and treatment of urolithiasis.

## Figures and Tables

**Table 1:** Personal characteristics of patients with urolithiasis and the controls (1:1 matched by age and sex, N = 70 pairs).

Variables	Controls	Urolithiasis	
	N (%)		*p*-value ^a^
Gender			
Male	56 (80.00)	56 (80.00)	
Female	14 (20.00)	14 (20.00)	1.000
Labor intensity			
Light	60 (85.71)	50 (71.43)	
Moderate	10 (14.29)	20 (28.57)	0.040
Fluid intake (mL/day)			
<1000	8 (11.43)	12 (17.14)	
1000–2000	35 (50.00)	38 (54.29)	0.528
>2000	27 (38.57)	20 (28.57)	0.191
Cigarette smoking			
No	44 (62.86)	39 (55.71)	
Yes	26 (37.14)	31 (44.29)	0.390
Alcohol drinking			
No	59 (84.29)	48 (68.57)	
Yes	11 (15.71)	22 (31.43)	0.029
Swimming (≥1 time/summer)			
No	68 (97.14)	62 (88.57)	
Yes	2 (2.86)	8 (11.43)	0.049
Meals at canteens			
Barely	45 (64.29)	28 (40.00)	
Frequently	25 (35.71)	42 (60.00)	0.004
Meals at restaurants			
Barely	44 (62.86)	25 (35.71)	
Frequently	26 (37.14)	45 (64.29)	0.001
	Mean ± SD		*p*-value ^b^
Age	48.95 ± 1.48	49.01 ± 1.50	0.595
BMI (kg/m^2^)	24.25 ± 0.41	24.45 ± 0.39	0.728
Indexes of kidney function			
Urinary pH	5.91 ± 0.09	6.05 ± 0.07	0.244
Serum urea nitrogen (mmol/L)	5.24 ± 0.16 *	5.38 ± 0.17	0.454
Serum creatinine (μmol/L)	79.31 ± 1.88 *	92.29 ± 4.00	<0.001
Serum uric acid (μmol/L)	339.3 ± 10.41 *	470.8 ± 102.6	0.029
Melamine and its analogues (ng/mL)			
Melamine	11.79 ± 1.53	7.33 ± 2.26	0.109
Ammeline	0.50 ± 0.12	0.37 ± 0.10	0.414
Ammelide	3.37 ± 0.31	3.10 ± 0.37	0.565
Cyanuric acid	12.43 ± 1.53	34.87 ± 3.99	<0.001

^a^*p*-value for chi-square test; ^b^*p*-value for paired *t*-test; SD, standard deviation. * The case group has 29 missing values on serum urea nitrogen and serum creatinine and 28 missing values on serum uric acid. Average value was applied to fill in the missing values.

**Table 2:** Personal characteristics in association with the risk of urolithiasis by conditional logistic regression models (1:1 matched by age and sex, N = 70 pairs).

Variables	Univariate		Multivariate *	
	OR (95%CI)	*p*-Value	OR (95%CI)	*p*-Value
Labor intensity				
Light	Reference		Reference	
Moderate	2.25 (0.98, 5.18)	0.056	1.63 (0.63, 7.31)	0.523
Fluid intake (mL/day)				
<1000	Reference		Reference	
1000–2000	0.72 (0.27, 1.89)	0.502	0.91 (0.12, 6.99)	0.927
>2000	0.40 (0.12, 1.32)	0.134	0.74 (0.07, 7.56)	0.801
Cigarette smoking				
No				
Yes	1.42 (0.68, 2.97)	0.356	1.71 (0.42, 6.93)	0.455
Alcohol drinking				
No	Reference		Reference	
Yes	2.57 (1.07, 6.16)	0.034	1.91 (0.44, 8.24)	0.385
Swimming (≥1 time/summer)				
No	Reference		Reference	
Yes	7.00 (0.86, 56.89)	0.069	5.66 (0.31, 102.16)	0.240
Meals at canteens				
Barely	Reference		Reference	
Frequently	3.13(1.41, 6.93)	0.005	1.92 (0.47, 7.79)	0.361
Meals at restaurants				
Barely	Reference		Reference	
Frequently	4.80 (1.83, 12.58)	0.001	5.71 (1.01, 32.31)	0.049
Age	1.13 (0.72, 1.76)	0.593	1.11 (0.48, 2.55)	0.812
BMI (kg/m^2^)	1.02 (0.92, 1.13)	0.726	0.94 (0.78, 1.13)	0.501
Indexes of kidney function				
Urinary pH	1.32 (0.83, 2.09)	0.246	1.17 (0.52, 2.60)	0.707
Serum urea nitrogen (mmol/L)	1.12 (0.84, 1.50)	0.452	1.12 (0.64, 1.95)	0.690
Serum creatinine (μmol/L)	1.06 (1.03, 1.10)	<0.001	1.07 (1.01, 1.14)	0.024
Serum uric acid (μmol/L)	1.01 (1.01, 1.02)	<0.001	1.01 (1.00, 1.02)	0.042
Melamine and its analogues				
Melamine	0.98 (0.96, 1.01)	0.130	0.98 (0.93, 1.02)	0.339
Ammeline	0.84 (0.56, 1.28)	0.418	0.98 (0.62, 1.56)	0.932
Ammelide	0.96 (0.84, 1.10)	0.565	0.54 (0.21, 1.38)	0.197
Cyanuric acid	1.12 (1.07, 1.18)	<0.001	1.11 (1.02, 1.21)	0.021

OR (95 CI%), odds ratio (95% confidence interval). * Adjusted by significant variables in Table 1, including labor intensity, fluid intake, alcohol-drinking behavior, swimming frequency in summer, frequently having meals at canteens, frequently having meals at restaurants, BMI, serum creatinine, and uric acid.

**Table 3:** Stratified concentration of CYA in association with the risk of urolithiasis by logistic regression models.

CYA Conc. (ng/mL)	Controls	Urolithiasis	Univariate		Multivariate *	
	N (%)	N (%)	OR (95%CI)	*p*-Value	OR (95%CI)	*p*-Value
Stratified by median						
<19.3	56 (80.00)	14 (20.00)	Reference		Reference	
≥19.3	14 (20.00)	56 (80.00)	16.00 (6.99, 36.63)	<0.001	24.36 (7.79, 76.21)	<0.001
Stratified by quartile						
<11.0	39 (55.71)	2 (2.86)	Reference		Reference	
11.0–19.7	19 (27.14)	14 (20.00)	14.37 (2.96, 69.75)	0.001	13.94 (2.35, 82.70)	0.004
19.7–32.5	8 (11.43)	25 (35.71)	60.94 (11.95, 310.65)	<0.001	83.69 (11.74, 596.62)	<0.001
32.5–271.3	4 (5.71)	29 (41.43)	141.38 (24.22, 825.11)	<0.001	118.65 (17.21, 817.97)	<0.001

* Adjusted by significant variables in Table 1, including labor intensity, fluid intake, alcohol-drinking behavior, swimming frequency in summer, frequently having meals at canteens, frequently having meals at restaurants, BMI, serum creatinine, and uric acid.

## Data Availability

The datasets of this study are available from the corresponding author on reasonable request.
